# The PIEPEAR Workflow: A Critical Care Ultrasound Based 7-Step Approach as a Standard Procedure to Manage Patients with Acute Cardiorespiratory Compromise, with Two Example Cases Presented

**DOI:** 10.1155/2018/4687346

**Published:** 2018-06-11

**Authors:** Wanhong Yin, Yi Li, Shouping Wang, Xueying Zeng, Yao Qin, Xiaoting Wang, Yangong Chao, Lina Zhang, Yan Kang, Chinese Critical Ultrasound Study Group (CCUSG)

**Affiliations:** ^1^Department of Critical Care Medicine, West China School of Medicine/West China Hospital, Sichuan University, 37 Guoxue Avenue, Chengdu 610041, China; ^2^Department of Critical Care Medicine, Peking Union Medical College Hospital, Peking Union Medical College, Chinese Academy of Medical Sciences, Beijing 100730, China; ^3^Department of Critical Care Medicine, The First Hospital of Tsinghua University, Beijing 100016, China; ^4^Department of Critical Care Medicine, Xiangya Hospital, Central South University, Changsha, Hunan 410008, China; ^5^West China Hospital, Sichuan University, China

## Abstract

Critical care ultrasound (CCUS) has been widely used as a useful tool to assist clinical judgement. The utilization should be integrated into clinical scenario and interact with other tests. No publication has reported this. We present a CCUS based “7-step approach” workflow—the PIEPEAR Workflow—which we had summarized and integrated our experience in CCUS and clinical practice into, and then we present two cases which we have applied the workflow into as examples. Step one is “problems emerged?” classifying the signs of the deterioration into two aspects: acute circulatory compromise and acute respiratory compromise. Step two is “information clear?” quickly summarizing the patient's medical history by three aspects. Step three is “focused exam launched”: (1) focused exam of the heart by five views: the assessment includes (1) fast and global assessment of the heart (heart glance) to identify cases that need immediate life-saving intervention and (2) assessing the inferior vena cava, right heart, diastolic and systolic function of left heart, and systematic vascular resistance to clarify the hemodynamics. (2) Lung ultrasound exam is performed to clarify the predominant pattern of the lung. Step four is “pathophysiologic changes reported.” The results of the focused ultrasound exam were integrated to conclude the pathophysiologic changes. Step five is “etiology explored” diagnosing the etiology by integrating Step two and Step four and searching for the source of infection, according to the clues extracted from the focused ultrasound exam; additional ultrasound exams or other tests should be applied if needed. Step six is “action” supporting the circulation and respiration sticking to Step four. Treat the etiologies according step five. Step seven is “recheck to adjust.” Repeat focused ultrasound and other tests to assess the response to treatment, adjust the treatment if needed, and confirm or correct the final diagnosis. With two cases as examples presented, we insist that applying CCUS with 7-step approach workflow is easy to follow and has theoretical advantages. The coming research on its value is expected.

## 1. Introduction

Critical care ultrasound has been widely used to examine patients from head to toe in the ICU [1–7]. Protocolized ultrasound examination is so important that with it the information of multiple organs could be integrated as a full picture to make us understand more about the patient, which is also the unique superiority of critical care ultrasound compared to the other imaging or nonimaging tests [8–13]. To date, there have been many studies on the protocols focusing on protocolized ultrasound examination to improve the accuracy of diagnosis and efficiency of critical care ultrasound utilization to guide a better treatment [14–28]. Several studies have shown better outcome when applying protocolized critical care ultrasound into the clinical diagnosis and therapy [29, 30]. However, these protocols are focusing on one aspect of those of hemodynamic assessment, respiratory disorder identification, trauma assessment, and so forth and ultrasound was the only tool to use [15, 16, 18, 23]. As a tool of critical care medicine, critical care ultrasound has very limited usage without integration with clinical information and other monitoring tools, and it is often required to be repeated dynamically. With a good integration with other clinical information, critical care ultrasound is a superior tool to guide the discovery of pathology deteriorations as well as searching for disease etiology, for example, the source of infection [31–33]. To our knowledge, there is no publication to describe a detailed workflow that can integrate the critical care ultrasound with the clinical information and other examinations to improve diagnosis, treatment, and patients' follow-up. The aim of this paper is to propose a novel “PIEPEAR” workflow: the ultrasound based 7-step approach workflow summarized from the our daily practice since 2014 in a 50 beds' critical care unit. Two cases are presented as examples.

## 2. The PIEPEAR Workflow: A Critical Care Ultrasound Based 7-Step Approach

The PIEPEAR Workflow is designed to be applied in the setting of acute clinical deterioration of circulation and respiratory and oriented by the clinical problem as problem based examination is one of the key features of critical care ultrasound [5]. The clinical questions give the strong aim of focusing the examination on the key views and variables of the patient, which makes critical care ultrasound more efficient and valuable to practice [5, 36–38]. The contents of the workflow were listed in Table 1. As it is the standard work procedure not a simple exam protocol, the contents are comprehensive but methodical.

Step one is “problems emerged?” thus classifying the signs of the deterioration into two aspects: acute circulatory compromise and acute respiratory compromise. For instance, if the patient presents with symptoms as heart rate increase/drop, hypotension, oliguria, acidosis, or increased requirement of vasopressor, the question would be “is the patient on acute circulatory collapse?”; if the patient presents with the symptoms as acute respiratory distress, decrease in oxygenation, increased dependence of ventilator, or patient-ventilator asynchrony, the question would be “is the patient on acute respiratory collapse?” The above problems have covered most of the cases that we may encounter in our daily work in an ICU setting. We focus on the circulation and respiration because collapse in circulation and/or respiration is the ultimate consequence of most of the severe disease etiologies [39, 40], and patients' acute deterioration in ICU is mostly noted by the symptoms and parameters alteration in circulation and respiration [5, 37, 41]. On the other hand, categorizing the problems either in circulation or in respiration not only makes critical care ultrasound easier to focus on the target organs but also facilitates the order for other relative clinical examinations.

Step two is “information clear?” quickly summarizing the patient's medical history by three aspects, namely, the basic cardiopulmonary function, the cardiopulmonary disorder on admission and the progress, and the current clinical manifestation and the lab variables of the patient's deterioration. This step is necessary because one cannot make the report accurately without individually concerning the basic cardiorespiratory function and the new change of the patient [42, 43]. For example, an enlarged right ventricle in a patient without basic heart disease often means potential acute disorders that the physician needs to find out and deal with the etiology as soon as possible, but it may be meaningless and commonly seen in a patient with chronic pulmonary disease [44, 45] like chronic obstructive pulmonary disease (COPD); an IVC of 2cm in diameter usually represents fluid overload; however, it can be normovolemia in patients with chronic right heart failure [42, 46, 47]. Besides, the new onset of symptoms, physical signs, and lab findings are also essential to explain the ultrasonic findings accurately. To save time, this step should be performed either in preparing the ultrasound examination or in the process of examination in the form of oral presentation on bedside by colleagues. It could also be initiated after the examination in case of an emergency that there is no time to perform this step before or during examination.

**Figure 1:**
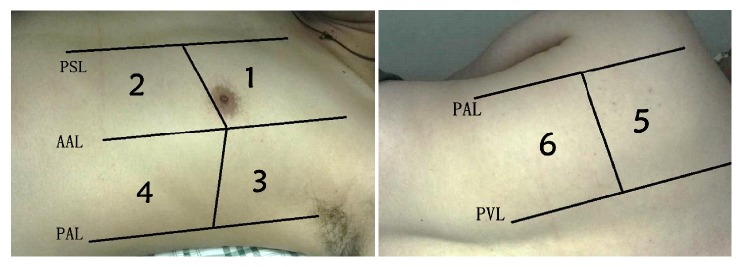
**Regions of lung ultrasound examination**. There are six examination regions on each side, delineated by parasternal line (PSL), anterior axillary line (AAL), posterior axillary line (PAL), and paravertebral line (PVL) [34, 35].

Step three is “focused exam launched”; thus launch the focused echocardiography and lung ultrasound no matter if there is a single system failure or there are both the circulation and respiration failure [10, 12, 41]. That is because several studies have shown that the combination of echocardiography and lung ultrasound discovered more insults and provided more information to facilitate diagnosis [8, 9]. Echocardiography is supposed to be the first part and is done as two steps, and the first step is the fast and global assessment of the heart (heart glance) and thus identifies cases that need immediate life-saving intervention by intensivists or cardiologist (listed in Table 1). It is crucial that such situations be identified as early as possible to facilitate an immediate treatment, where showing the unique advantage of the critical care ultrasound compared to the other tools we have in the ICU [48]. The second step is assessing the circulatory system in the following order: (1) assessing the IVC, which aims to identify the volume status and fluid responsiveness [49]; (2) evaluating the right heart to identify the acute right heart dysfunction that may harm the output of left side of the heart or cause a false positive monitoring which may mislead the treatment [50–54]; (3) assessing the function of diastole and systole of left heart; (4) deducing systemic vascular resistance. The measuring of pulmonary artery occlusion pressure (PAOP) should be included into the assessment of the diastolic function to alert the hydrostatic pulmonary edema and the risk to initiate fluid therapy [55–57]. The systolic function would be evaluated with eyeballing to identify the regional wall motional abnormalities and then categorize the function [58–60]; and the systemic vascular resistance is roughly deduced with the above variables or calculated with MAP and SV accurately, in which situation SV should be measured [61]. Echo should always be followed by lung ultrasound exam. The twelve-region method (Figure 1), in which the chest is divided into twelve examination regions, is recommended rather than the BLUE protocol, since more information would be generated despite more time consumption [34, 35]. Though some papers indicate that the consolidation and B lines in the 5th and 6th regions bilaterally might be meaningless in ICU settings, we recommend involving them as they might be helpful in some cases [62, 63]. After excluding the respiratory emergency (i.e., unstable pneumothorax), the main task for lung ultrasound exam is to find the predominant profile of the lung, which is valuable to guide the supportive therapy and indicate the clues for diagnosing [36, 37].

Step four is “pathophysiologic changes reported” thus integrating the ultrasound and clinical information by asking the question and reporting it: what do we know about the pathophysiologic disorders of the circulation and respiration? The answer is the key of supportive treatments of the circulation and respiration [41, 64, 65] and the clues to instruct the etiology exploration.

Step five is “etiology explored.” Diagnose the etiology by integrating the above four steps; search for the source of infection according the clues extracted from the focused ultrasound exam; additional ultrasound exams or other tests should applied if needed. The clues are listed as examples: acute cor pulmonale indicates pulmonary embolism, unreasonable ventilation setting, or severe mismatch of the ventilation and flow representing ARDS [45]; acute increase in PAOP derives from decrease in systolic function, left side valve insufficiency, hypervolemia, or decreased myocardium compliance [66]; diffuse sonointerstitial syndrome (SIS) indicates hypervolemic pulmonary edema, cardiogenic pulmonary edema, leakage pulmonary edema, acute pneumonitis, pulmonary alveolar proteinosis, and chronic pulmonary fibrosis [6, 37, 67]; consolidation with shred sign in lung ultrasound indicates pneumonia [68–70]; hypoechoic yet heterogeneous at plural cavity indicates hemothorax or pyothorax [32, 33]; echogenic dots in free fluid indicate abscessus [33, 71]; echogenic dots in physiology cavity indicate infection [33, 71] and so forth. With the clues above, we can clearly know what to do next and the diagnosis procedure can be efficient. Guiding the diagnosis of the etiology and source searching is a remarkable competence of critical care ultrasound [32]. However, the other exams are strongly required in the diagnosis procedure, and critical care ultrasound is more like a guide. This step is very essential to the patient outcome [31, 72].

Step six is “action.” Support the circulation and respiration sticking to step four; treat the etiologies according Step five.

Step seven is “recheck to adjust.” Repeat focused ultrasound and other tests to assess the response to treatment, adjust the treatment if needed, and confirm or correct the final diagnosis. The judging of the response includes two aspects: the returning to normality of the abnormal ultrasonic findings which may also contribute to confirming the reliability of the ultrasound variables and the improvement of the patient such as the stabilization of vital signs, increase of oxygenation, decrease of lactate level, and so forth [73, 74].

## 3. Case 1 Presentation 

A 4-year-old boy was admitted to pediatric department because of newly occurred hypertension as status posterior right heminephrectomy of neuroblastoma. Computed tomography angiography (CTA) scan revealed renal artery severe stenosis and right kidney atrophy. He had undergone Transcatheter Arterial Embolization of right renal artery 4 days ago because of the refractory hypertension. He was stable after the surgery and transfused 1 unit of packed red blood cells due to anemia. Five hours later, he became anxious and breathless, spitted frothy sputum, and then suffered an episode of cardiac arrest. After being intubated and 20 minutes' CPR, he underwent restoration of spontaneous circulation (ROSC). The attending physician treated him with cortisone as transfusion related acute lung injury (TRALI) was suspected. Then the patient was transferred to the ICU to receive respiratory support and further treatment. At presentation, he had a heart rate of 160 times/min and blood pressure of 150/111mmHg without any vasoactive drugs. A lot of flesh-colored aqueous sputum was spurred out of endotracheal tube. Tidal volume is only about 30ml on invasive ventilation with PI 15cmH_2_O and PEEP 10 cmH_2_O (PCV mode). Before he arrived to the ICU, the patient received manual ventilation with balloon and sputum suction constantly for 1 hour. The lung was very stiff and hard to inflate by balloon. Arterial blood analysis showed pH 6.7, PO_2_ 56mmHg, PCO_2_ 28mmHg, lactate 16 mmol/l, and BE -30. The FiO_2_ was 100%. There was no urine output in the first hour. We performed critical care ultrasound using the 7-step approach workflow at that time to make the puzzle clear (Table 2).

## 4. Case 2 Presentation

A 61-year-old male patient was admitted to the liver surgery department because of discovering liver mass for 6 days. The alpha-fetoprotein (AFP) was 1009 ng/ml, and liver contrast CT scan indicated hepatic cell cancer in the right lobe. As a generally healthy status before surgery, the patient received ALTPS surgery. 20 days later, he got fever and abdominal pain and developed shock as well as hypoxia in hours. He was intubated and treated with fluid resuscitation and norepinephrine (1.8 mcg/Kg.min) and then transferred to the ICU. Auxiliary examination showed WBC 0.63×10^9^/L, PLT 7×10^9^/L, and PCT 45.88 ng/ml; bedside ultrasound was ordered and ascites were found. The doctors cultured and drained the ascites and treated him with Imipenem and Vancomycin. Then they ordered abdominal CT and it reveals signs of necrosis of right lobe of the liver. Later, the patient suffered the second surgery to remove the right half of the liver. Culture of ascites reports* Escherichia coli*. After three days, the patient got better. No fever existed and the norepinephrine had been decreased to 0.4 mcg/Kg.min, and urine output had been maintained at 2000–2500ml per day. Two days later the patient had fever again, with the highest temperature of 38.8°C, as well as an increase of norepinephrine from 0.4 mcg/Kg.min to 2.0 mcg/Kg.min, deterioration of liver function, coagulation, and oxygenation. Arterial blood gas test showed pH 6.988, PaO_2_/FIO_2_ 154, PaCO_2_ 147.7mmHg, BE -19 mmol/L, and lactate 9.7 mmol/L. We performed critical care ultrasound using the 7-step approach workflow at that time to make the puzzle clear (Table 3).

## 5. Discussion

Critical care ultrasound has been widely used as a reliable tool in a whole setting of critical care practice [1–3, 48, 75]. As noninvasive, visible point of care handling both monitoring and diagnosis, it has the advantages that any other single tool can not have [48]. In an intensivist's hand, critical care ultrasound can not only visualize the organ structure as well as the physiopathological changes but also find out the clues to instruct the diagnosis and source searching. The exam procedure is flexible either focusing on cardiorespiratory assessment or supplementing additional examination according the requirement. Critical care ultrasound is competent for both static monitoring and dynamic assessment for titrating or adjusting the treatment. Above all, critical care ultrasound is an excellent tool with which we can integrate the critical care theory and practice and cooperate with other examination methods. To date, a lot of studies focus on the protocols integrating multiorgan's ultrasonic information to contribute to the diagnosis and treatment [14–28]. Such protocols all focus on single aspect. Although these may be valuable [76], the imperfection of those protocols is that they only use the ultrasound to answer one specific question regarding physiopathological disorders rather than to solve a clinical case. The ultrasound should be integrated within the medical history and the critical care theory and cooperate with other tools dynamically to handle the whole care course. One key feature in the critical care setting is the treatment should cover both supportive treatment and etiology therapy and should be conducted dynamically and continuously [77].

In our opinion, the PIEPEAR Workflow has promoted the value of critical care ultrasound. After Step one “problems emerged?” we have information about what happened and what to do next. Then all the efforts can be focused on the key points, such as circulation and respiration. What the Step two does is make the report and further treatment plan more accurate. The ultrasound exam plan in Step three draws lessons from the critical care theory and promotes the advantages of critical care ultrasound. Firstly, in case of deterioration, based on the critical thinking, the most important thing is to verify or exclude the critical cases that could cause an immediate cardiac arrest such as severe shock and tamponade. Just as rescuing would be the first thing to do when encountering septic shock according to the four-stage treatment by Vincent et al. [78], we need to first pull the dying patients back against the collapse. So when concerning the circulation the heart glance is the first of the echo examination. For instance, when encountering the severe shock or bradycardia, we should do everything to find out if there are emergencies in cardiovascular area such as tamponade, severe hypovolemia, and massive pulmonary embolism; also lung would be checked to make sure that no tension pneumothorax exists as it would also harm circulation. The above reveals the unique competence of critical care ultrasound compared with other tools [25, 26, 79]. Secondly, unlike other protocols that focus only on the function and output of LV, the 7-step approach seeks details of the RV, LV systolic, LV diastolic, and afterload separately. With detailed assessment of the hemodynamics, we can exactly classify the patients into different types to intervene individually, which is important to achieve a better outcome [50, 61, 80–83]. Take the valvular heart disease as an example; the treatment is different when the valve insufficiency is attributed to the circulation compromise [84, 85]. Judging whether it is suitable for operation and also monitoring the transvalvular pressure gradient, regurgitation, and output are considered to be more important. The third, the lung ultrasound exam shows the predominant pattern to deduce lung pathology, which can guide support care and instruct the etiology diagnosis [6, 37]. The last four steps highlight the beneficial side of the workflow as follows: (1) the workflow facilitates both supportive care and etiology therapy simultaneously, owing to critical care ultrasound integrating with clinical information and other exams. This is valuable as neither support care nor etiology therapy works without each other, and, just like the treatment of septic shock, fluid resuscitation cannot improve outcome without early administration of antibiotics and/or drainage of the infection source [31, 72]. (2) The other tools are involved to cooperate with critical care ultrasound which means the workflow draws more advantages to work. (3) The workflow consists of the dynamic monitoring to feedback and correction, also titrating the goal achievement, which meant to enlarge the effect and decrease the treating associated injury.

The focused cardiorespiratory ultrasound has the main role and the other parts' ultrasound exam works as a supplement when needed. For example, eFAST is to be performed when the traumatic patient represents hypovolemia in ultrasound assessment [23, 86]. A patient that suffered acute cor pulmonale (ACP) is thought to add an examination of deep venous thrombosis (DVT) [18]. Such design is regarded to be more effectible.

In case 1, the patient was previously diagnosed with transfusion related acute lung injury (TRALI). The diuretic treatment was criticized as a big part of the patients of TRALI, which were hypovolemia due to the fluid loss. Furthermore, the doctors usually choose resuscitation in case of compromised circulation [87, 88]. Our patient was in such puzzle. However, when we applied the seven-step approach on this case the puzzle was solved. The diuretic treatment initiated as the hypervolemia had been proved by the filled heart chamber and distended IVC, and the hypervolemic pulmonary edema was proved by multiple B lines and elevated PAOP. After we initiated the diuresis treatment, B lines decreased and there was no blood pressure drop despite the diuresis, which demonstrated that the previous judgement was correct and the ultrasound report was reliable. But TRALI could not be excluded yet, so the other tests especially the albumin in Bronchoalveolar Lavage Fluid (BALF) should be ordered [87]. The patient was finally diagnosed and successfully recovered. This is a good example for the workflow.

In case 2, in the PIEPEAR Workflow, the patient presented with severe hypovolemia, and then fluid resuscitation and goal-directed therapy were initiated. Further, the occurrence of hyperdynamic shock combined with the medical history indicated septic shock, which launched the diagnosis flow of septic shock, and antibiotic treatment, source searching, and blood culture. The critical care ultrasound evidence of massive consultation could not diagnose pneumonia but when combined with the medical history, lab measurements, and other variables of organ function, the diagnosis was confirmed. Notice that the critical care ultrasound gave the clues to initiate and lead the whole procedure of diagnosis and treatment [37]. In the rechecking after 4 hours, the patient was found to be nonresponsive and there was an increase of PAOP and lung water [57], owing to which the fluid prescription was ceased in time. These were all driven by the seven-step approach workflow.

We provide these two typical cases to show how the 7-step approach worked. Keep in mind that not all cases are appropriate for the workflow, and critical care ultrasound is not a “magic bullet” but a key. However, the way the critical care ultrasound works in the PIEPEAR Workflow does open a new door for treating challenge cases or clinical dilemma in critical care setting. For now, this approach has not been proved by prospectively designed and randomized controlled trials. However, it is a summary of experiences based on daily critical care practice, and the trial to confirm its efficacy can be designed in the future based on current data base.

It is important to note that the application of critical care ultrasound should not be restricted in a firmed protocol, and the flexibility to use it by the physician in front of the individual cases is essential. The workflow we provide is more like an orientation to guide daily clinical work and facilitate clinical logistic, especially for the new users of critical care ultrasound.

## 6. Conclusions

Applying critical care ultrasound with the PIEPEAR Workflow is easy to follow and has shown its advantages, and the coming research on its value is expected.

## Figures and Tables

**Figure 2:**
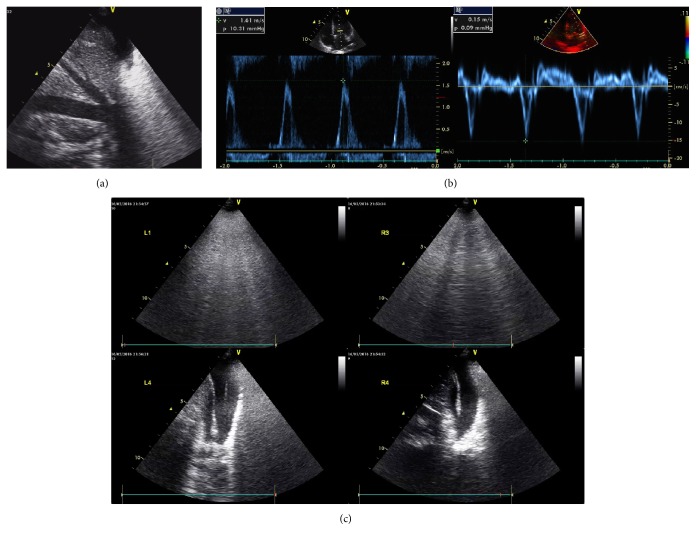
**Abnormal findings of the Focused Ultrasound Assessment on admission of case **1. (a) IVC exam. Fixed and enlarged IVC and hepatic vein represented no fluid responsiveness and maybe hypervolemia. (b) Assessment of diastole. Restrictive diastolic dysfunction was presented and PAOP estimated by E/e' was increased. (c) Lung ultrasound exam. Bilateral multiple B lines, with posterior atelectasis and plural effusion, indicate diffuse sonographic interstitial syndrome.

**Figure 3:**
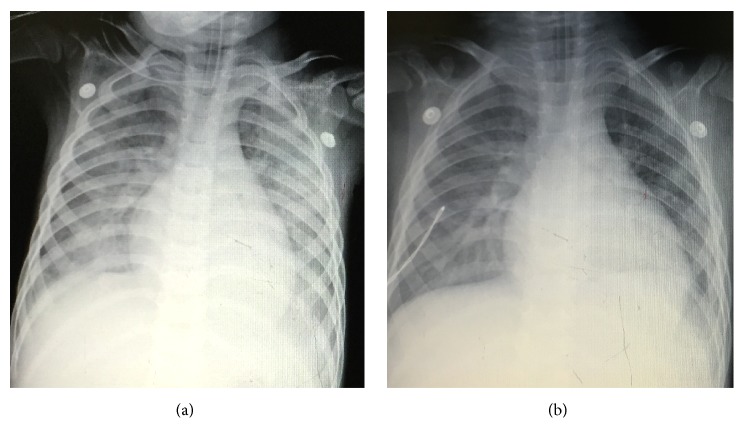
**Chest X-ray exam on admission and the second day of case **1. (a) On admission, bilateral symmetrical infiltration response for pulmonary edema was revealed. (b) The second day, bilateral pulmonary edema was obviously decreased.

**Figure 4:**
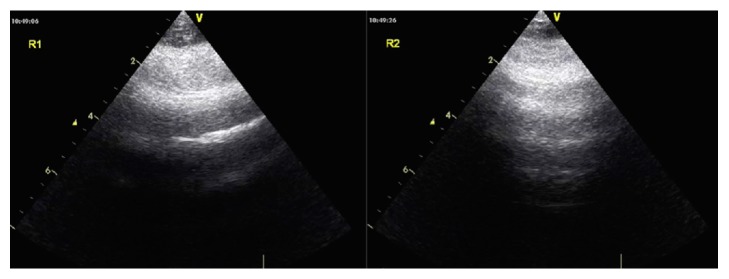
**Part of lung ultrasound in the repeated ultrasound exam in the next day**. Bilateral A-lines in bilateral 1st regions indicate pulmonary edema was obviously decreased.

**Figure 5:**
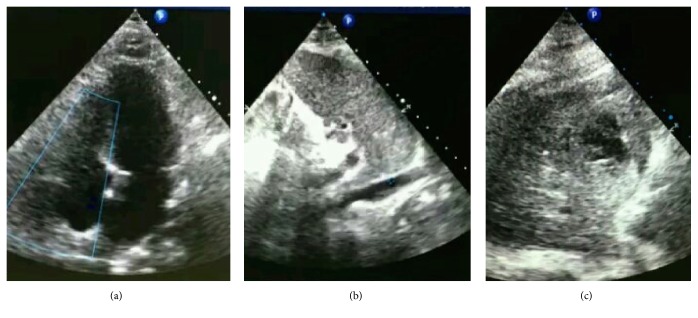
**Abnormal findings of the Focused Ultrasound Assessment on admission of case **2. (a) Heart browse. No circumstances that need immediate life-saving intervention or cardiologist emergency consultation; left ventricle apex balloon. (b) IVC exam. The diameter <1cm representing hypovolemia. (c) Lung ultrasound exam. Right lung massive consolidation (from the 2nd right region to the 6th right region).

**Figure 6:**
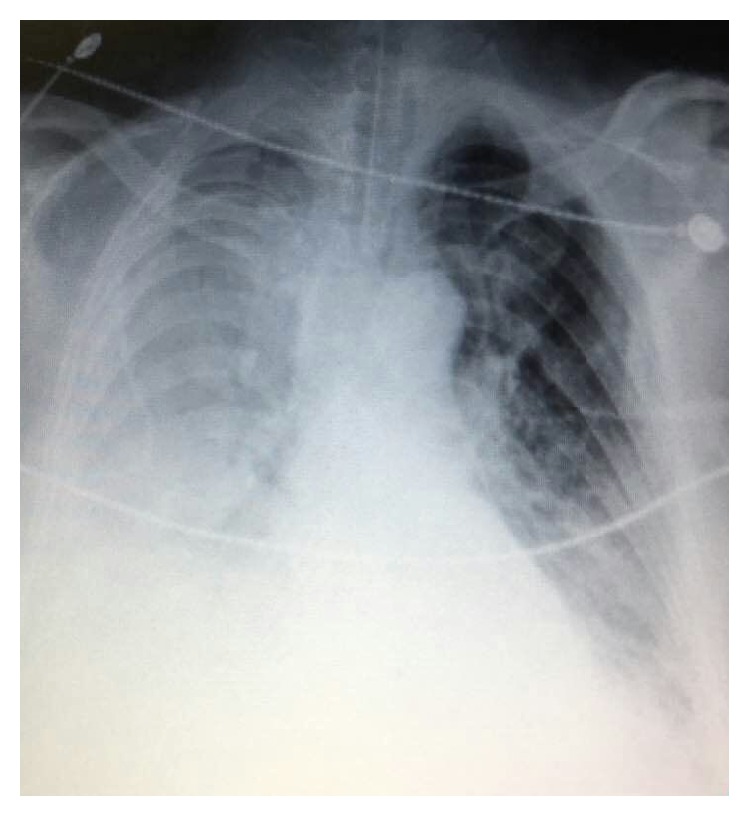
**Chest X-ray exam of case **2. Large hyperdensity in right lung showing right lung consolidation, which represented right lung pneumonia in this case.

**Table 1:** The critical care ultrasound based PIEPEAR workflow.

Outlines	Rationale	Tale
1. Problems emerged?	The signs of the deterioration should be classified into two aspects: acute circulatory compromise and acute respiratory compromise	Heart rate increase/drop, hypotension, oliguria, acidosis, increased requirement of vasopressor or other symptoms would be defined as **acute circulatory compromise**
		Acute respiratory distress, decrease in oxygenation, increased dependence of ventilator, patient-ventilator asynchrony, or other symptoms would be defined as **acute respiratory compromise**

2. Information clear?	Quickly summarize the patient's medical history by three aspects	(1)Any dysfunction of heart and lung caused by basic or chronic diseases?
		(2)The main dysfunction of circulation and respiration when admitted and its progress?
		(3)The current clinical manifestation and the lab variables of the patient's deterioration?

3. Focused exam launched	Focuses exam of the heart by five views, and the contents are listed in the right cell	**(1)Heart glance**: fast and global assessment of the heart to identify cases that need immediate life-saving intervention by intensivists or cardiologist, such as tamponade, acute cor pulmonale (pulmonary embolism/tension pneumothorax), ACS, catastrophic valve pathology (AIE/valve failure on chronic valve diseases), and aortic dissection.** (2) IVC exam**: static volume status, fluid responsiveness.** (3)RV exam**: identify the acute right heart dysfunction that may harm the output of left side of the heart or cause a false positive monitoring what may mislead the treatment.** (4)Diastole of left heart**: assesses PAOP increase that could alert the hydrostatic pulmonary edema and the risk to fluid initiation.** (5) Systole of left heart**: use eyeballing to classify the contraction into 3 categories: hyperkinesis, normal, and abnormal, then identify the abnormality as disseminated or regional wall motion abnormalities, and categorize the former as mild hypokinesis, moderate hypokinesis, and severe hypokinesis.** (6) Afterload**: deduce the afterload with the above indexes or calculate with MAP and SV accurately
	Divide the chest wall into 12 exam regions, ultrasonic pattern of each region should be integrated to conclude the overall profile of lung pathology	Identify each region as the following patterns: A pattern-lines associated or not with lung sliding; B pattern: three or more isolate B lines within a scan view; C pattern: consolidation or atelectasis; PE: intrapleura anechoic hypoechoic collection zone. Each pattern should include detailed information. A pattern would be detailed for lung sliding, lung pulse, and lung point; B pattern would be detailed for regularly spaced or irregularly spaced, normal or abnormal pleura; C pattern would be detailed for the morphology, regular or irregular margins, static or dynamic bronchograms; PE would be detailed for strength of the echo, separate or not

4. Pathophysiologic changes reported	The results of the focused ultrasound exam were integrated to conclude the pathophysiologic changes	The supportive treatment would be the basis of the pathophysiologic changes

5. Etiology explored	Diagnosis the etiology by integrating step two and step four; search for the source of infection, according the clues extracted from the focused ultrasound exam; additional ultrasound exams or other tests should applied if needed	(1) Some of the ultrasonic clues that may contribute to guiding the diagnosis: -Acute cor pulmonale may indicate pulmonary embolism, unreasonable ventilation setting, or severe mismatch of the ventilation and flow representing ARDS; acute increase in PAOP derives from decrease in systolic function, left side valve insufficiency, hypervolemia, or decreased myocardium compliance; diffuse SIS may indicate hypervolemic pulmonary edema, cardiogenic pulmonary edema, leakage pulmonary edema, acute pneumonitis, pulmonary alveolar proteinosis, and chronic pulmonary fibrosis; consolidation with shred sign in lung ultrasound may indicate pneumonia; hypoechoic yet heterogeneous collections at plural cavity indicate hemothorax or pyothorax; echogenic dots in physiology cavity indicate infection, etc. (2) Additional ultrasonography may be LVOT-VTI, FAST, airway, diaphragm, etc. (3) Other examinations include blood biochemical examination, CT, PICCO, etc.

6. Action	Support the circulation and respiration sticking to step four	Carry out supportive and other relevant treatments for the circulation and respiration guided by the findings of the pathophysiologic changes in Step four
	Treat the etiologies according step five	Carry out the therapy of the etiology (antibiotic, drainage of infection source, etc.) guided by the results of Step five

7. Recheck to adjust	Repeat focused ultrasound and other test to assess the response to treatment, adjust the treatment if needed, and confirm or correct the final diagnosis	Repeat ultrasonography in a case-by-case determined time frame to see whether the indexes get better or not Recheck the clinical information of the patient (vital signs, uop, lactate, ABG, etc.) Follow up with the patient and the clinical data, order further test or treatment if needed, and summarize the whole care process to achieve the final diagnosis

ACS: Acute Coronary Syndrome; AIE: acute infective endocarditis; IVC: Inferior vena cava; RV: right ventricle; LV: left ventricle: left ventricle; PAOP: pulmonary artery occlusion pressure; MAP: mean arterial pressure; SV: stroke volume; LUS: lung ultrasound score; LVOT-VTI: left ventricular outflow tract-Velocity Time Integral; FAST: focused assessment with sonography for trauma; CT: Computed Tomography; ABG: arterial blood gases; SIS: Sonointerstitial syndrome.

**Table 2:** Applying PIEPEAR workflow to case 1.

Outlines	Application
1. Problems emerged?	Acute circulatory compromise emerged—severe dyspnea+abundant flesh-colored endotracheal secretions
	Acute respiratory compromise emerged—cardiac arrest +heart rate increase+oliguria+elevated lactate

2. Information clear?	(1) No evidence of dysfunction of heart and lung before admission
	(2) Stable after surgery
	(3) Newly presented anxiety and dyspnea after transfusion and cardiac arrest, awake after 20 min's CPR, ABG showed severe hypoxia and extremely hypercapnia after intubated

3. Focused exam launched	**Heart browse**: no circumstances that need immediate life-saving intervention or cardiologist emergency consultation, no signs of valvular diseases **IVC exam**: no fluid responsiveness, maybe hypervolemia because of no collapse during respiration, and hepatic vein enlarged (Figure 2(a)) **RV exam**: no right ventricular failure that may harm the function of left ventricle or misleading the therapy **Diastole of left heart**: Restrictive diastolic dysfunction was presented and PAOP estimated by E/e' was increased(Figure 2(b)) **Systole of left heart**: a filling cavity, mild to moderate dysfunction, no RWMA **Afterload**: increased
	Bilateral inferior and lateral B pattern, with posterior atelectasis and plural effusion, indicate diffuse sonographic interstitial syndrome (Figure 2(c))

4. Pathophysiologic changes reported	Pulmonary edema, hypervolemic and cardiogenic as CCUS indicates; increased-permeability pulmonary edema may also be suspected when involved with the history of transfusion

5. Etiology explored	(1) (1) Acute hypervolemic and cardiogenic pulmonary edema (2)Transfusion-related acute lung injury? (2) Test BNP, WBC, test the albumin of the endotracheal secretions, CXR when possible, repeat ABG are needed

6. Action	Diuresis to eliminate extra fluid, PEEP increase to reaerate the alveolar, continuing draining the secretions
	Continue cortisone, and further using blood products was prohibited

7. Recheck to adjust	PAOP and B lines decreased after 200ml urine in two hours Two hours later, oxygen improved as well as the internal environment (PO2 increased from 56 to 125mmHg, Lac decreased from 16 to 11.8 mmol/l, pro-BNP>35000 pg/ml, CXR revealed bilateral symmetrical infiltration, Figure 3(a)) Confirm: acute hypervolemic edema existed; the current treatment should be continued. Ratio of protein in ETA to protein in plasma was 0.8 (31.7/39.6). The strength of ventilator also decreased the next day. Lung ultrasound showed bilateral A-lines(Figure 4) and the second day's CXR revealed that bilateral pulmonary edema was obviously decreased, as shown in Figure 3(b). Final diagnosis: TRALI associated hypervolemic pulmonary edema.

CPR: cardiopulmonary resuscitation; ABG: arterial blood gases; IVC: Inferior vena cava; RV: right ventricle; PAOP: pulmonary artery occlusion pressure; E/e': early diastolic transmitral velocity to early mitral annulus diastolic velocity ratio; RWMA: regional wall motion abnormality; CCUS: critical care ultrasound; PEEP: Positive End Expiratory Pressure; BNP: brain natriuretic peptide; WBC: white blood cell; CXR: chest X ray; ETA: Endotracheal aspiration; TRALI: transfusion related acute lung injury.

**Table 3:** Applying PIEPEAR workflow to case 2.

Outlines	Application
1. Problems emerged?	Acute circulatory compromise emerged—hypotension+heart rate increase+oliguria+norepinephrine increase
	Acute respiratory compromise emerged—severe dyspnea+ extremely hypercapnia

2. Information clear?	(1) No evidence of dysfunction of heart and lung before admission
	(2) Septic shock when admitted to ICU, complicated intra-abdominal infections with *Escherichia coli* as the pathogen. After drainage of ascites, antibiotic therapy, fluid resuscitation, and other supportive treatments, the patient improved, presented as normal temperature, decreasing norepinephrine and normal urine output, etc.
	(3) Newly presented fever again, with the highest temperature of 38.8°C, as well as increasing norepinephrine to maintain blood pressure, deterioration of liver function, coagulation, and oxygenation. ABG analysis demonstrated the following: pH 6.988; PaO2 46.3mmHg with a FiO2 0.3 (PaO2 / FiO2 ratio of 154, PaCO2 147.7mmHg, BE -19mmol/L and lactate 9.70mmol/L.

3. Focused exam launched	Heart browse: no circumstances that need immediate life-saving intervention or cardiologist emergency consultation, mild to moderate tricuspid valve regurgitation, and left ventricle apex balloon (Figure 5(a)) IVC exam: hypovolemia as IVC diameter <1cm(Figure 5(b)), fluid responsiveness as dIVC>18% RV exam: no right ventricular failure that may harm the function of left ventricle or misleading the therapy Diastole of left heart: no evidence of diastolic dysfunction and PAOP elevated Systole of left heart: hyperdynamic, mild decrease in apex contraction Afterload: severely decreased
	Right lung massive consolidation (from the 2nd right region to the 6th right region, Figure 5(c))

4. Pathophysiologic changes reported	Hypovolemia with fluid responsiveness, severe decreased systemic vascular resistance which indicate hyperdynamic shock; acute respiratory failure caused by major consolidation and mismatch of the ventilation and blood flow

5. Etiology explored	Hospital acquired pneumonia? Septic shock? WBC, PCT, lactate, blood and ETA culture, PICCO, sonography for the abdomen are needed

6. Action	Fluid resuscitation guided by PICCO and CCUS; norepinephrine titration to MAP goal, use intravenous hydrocortisone if not achievable; monitoring lactate clearance and urine output to adjust above measures; titrate PEEP, recruitment the lung if it could be, deep sedation with neuromuscular blocking drugs, lung protect. If need, consider ECMO.
	Administrate broad-spectrum antibiotics, as treated sufficiently for *Escherichia coli* previously, drugs should aim at carbapenem-resistant acinetobacter and MRSA, as well as fungi. Drain the ETA, and search other sources of the patient if possible.

7. Recheck to adjust	Reexamination of CCUS after nearly four hours revealed no fluid responsiveness any more, massive consolidation in right lung and multiple B lines in left lung, PAOP elevated according to E/e'. PICCO reveals extremely low SVR despite high dose of norepinephrine, high EVLWI (PCCI 6.24 L/min/m2, GEDI 742 ml/m2, PPV 7 %, SVRI 522 dyn·s·cm-5·m2, EVLWI 26ml/kg). Adjustment: fluid resuscitation should be discontinued as no responsiveness and high risk of pulmonary edema. Blood culture reports carbapenem-resistant Acinetobacter baumannii (CRAB) CXR showed large hyperdensity in right lung which represented consolidation (Figure 6) **Final diagnosis: Hospital acquired pneumonia, septic shock.**

ABG: arterial blood gases; IVC: Inferior vena cava; dIVC: distention index of Inferior vena cava; RV: right ventricle; LUS: lung ultrasound score; WBC: white blood cell; PCT: procalcitonin; ETA: Endotracheal aspiration; CCUS: Critical care ultrasound; MAP: mean arterial pressure; PEEP: Positive End Expiratory Pressure; ECMO: extracorporeal membrane oxygenation; MRSA: Methicillin-resistant Staphylococcus aureus; PAOP: pulmonary artery occlusion pressure; E/e': early diastolic transmitral velocity to early mitral annulus diastolic velocity ratio; SVR: systemic vascular resistance; EVLWI: extra-vascular lung water index; PCCI: pulse contour cardio output index; GEDI: Global End-Diastolic volume Index; PPV: pulse pressure variation; SVRI: systemic vascular resistance index; CRAB: carbapenem-resistant Acinetobacter baumannii.

## Abbreviations

CCUS: Critical care ultrasound
IVC: Inferior vena cava
FALLS: Fluid administration limited by lung sonography
FATE: Focused assessed transthoracic echocardiography
FAST: Focused assessment with sonography for trauma
CCUE: Critical care ultrasonic examination
GDE: Goal-directed echocardiography
RUSH: Rapid ultrasound for shock and hypotension
RV: Right ventricle
LV: Left ventricle
ACS: Acute Coronary Syndrome
AIE: Acute infective endocarditis
COPD: Chronic obstructive pulmonary disease
PAOP: Pulmonary artery occlusion pressure
E/e': Early diastolic transmitral velocity to early mitral annulus diastolic velocity ratio
ARDS: Acute respiratory distress syndrome
LUS: Lung ultrasound score
EF: Ejection fraction
MAP: Mean arterial pressure
CT: Computed Tomography
ABG: Arterial blood gases
SIS: Sonointerstitial syndrome
ACP: Acute cor pulmonale
CTA: Computed tomography angiography
CPR: Cardiopulmonary resuscitation
ROSC: Restoration of spontaneous circulation
TRALI: Transfusion related acute lung injury
PCV: Pressure control ventilation
ETA: Endotracheal aspiration
SV: Stroke volume
LVOT-VTI: Left ventricular outflow tract-Velocity Time Integral
RWMA: Regional wall motion abnormality
PEEP: Positive End Expiratory Pressure
BNP: Brain natriuretic peptide
WBC: White blood cell
CXR: Chest X-ray
ETA: Endotracheal aspiration
dIVC: Distention index of Inferior vena cava
AFP: Alpha-fetoprotein
PCT: Procalcitonin
ECMO: Extracorporeal membrane oxygenation
MRSA: Methicillin-resistant Staphylococcus aureus
SVR: Systemic vascular resistance
EVLWI: Extravascular lung water index
PCCI: Pulse contour cardiac output index
GEDI: Global End-Diastolic volume Index
PPV: Pulse pressure variation
SVRI: Systemic vascular resistance index
CRAB: Carbapenem-resistant* Acinetobacter baumannii*
DVT: Deep venous thrombosis
BALF: Bronchoalveolar Lavage Fluid.
